# Crystal Structure and Functional Characterization of an Esterase (*Ea*EST) from *Exiguobacterium antarcticum*

**DOI:** 10.1371/journal.pone.0169540

**Published:** 2017-01-26

**Authors:** Chang Woo Lee, Sena Kwon, Sun-Ha Park, Boo-Young Kim, Wanki Yoo, Bum Han Ryu, Han-Woo Kim, Seung Chul Shin, Sunghwan Kim, Hyun Park, T. Doohun Kim, Jun Hyuck Lee

**Affiliations:** 1 Unit of Polar Genomics, Korea Polar Research Institute, Incheon, Republic of Korea; 2 Department of Polar Sciences, University of Science and Technology, Incheon, Republic of Korea; 3 Department of Chemistry, College of Natural Science, Sookmyung Women’s University, Seoul, Korea; 4 New Drug Development Center, Daegu-Gyeongpuk Medical Innovation Foundation, Daegu, Republic of Korea; Russian Academy of Medical Sciences, RUSSIAN FEDERATION

## Abstract

A novel microbial esterase, *Ea*EST, from a psychrophilic bacterium *Exiguobacterium antarcticum* B7, was identified and characterized. To our knowledge, this is the first report describing structural analysis and biochemical characterization of an esterase isolated from the genus *Exiguobacterium*. Crystal structure of *Ea*EST, determined at a resolution of 1.9 Å, showed that the enzyme has a canonical α/β hydrolase fold with an α-helical cap domain and a catalytic triad consisting of Ser96, Asp220, and His248. Interestingly, the active site of the structure of *Ea*EST is occupied by a peracetate molecule, which is the product of perhydrolysis of acetate. This result suggests that *Ea*EST may have perhydrolase activity. The activity assay showed that *Ea*EST has significant perhydrolase and esterase activity with respect to short-chain p-nitrophenyl esters (≤C8), naphthyl derivatives, phenyl acetate, and glyceryl tributyrate. However, the S96A single mutant had low esterase and perhydrolase activity. Moreover, the L27A mutant showed low levels of protein expression and solubility as well as preference for different substrates. On conducting an enantioselectivity analysis using R- and S-methyl-3-hydroxy-2-methylpropionate, a preference for R-enantiomers was observed. Surprisingly, immobilized *Ea*EST was found to not only retain 200% of its initial activity after incubation for 1 h at 80°C, but also retained more than 60% of its initial activity after 20 cycles of reutilization. This research will serve as basis for future engineering of this esterase for biotechnological and industrial applications.

## Introduction

Esterases (E.C. 3.1.1.X) are members of the *α*/*β*-hydrolase family that catalyze the hydrolysis of a variety of substrates containing ester linkages, such as aryl esters, acylglycerols, and carboxylic esters. These enzymes are widely distributed in bacteria, fungi, plants, insects, and animals, many of which are thought to play important physiological roles in lipid metabolism and detoxification of xenobiotics [1–5]. Carboxylesterases (carboxylester hydrolases, E.C. 3.1.1.1) and lipases (triacylglycerol acylhydrolases, E.C. 3.1.1.3) from microbial origin have increasingly gained interest in research, and a considerable number of novel enzymes have been discovered and characterized [6–8]. Many of them show substrate promiscuity and high regio- and stereoselectivity, as well as stability, in organic solvents; this makes them attractive biocatalysts for industrial processes including food modification, detergent formulation, and synthesis of fine chemicals and pharmaceuticals [9, 10].

Several crystal structures of microbial esterases have been described recently, such as those from the *Bacillus subtilis* strain 168, *B*. *subtilis* Thai I-8, and *Rhodopseudomonas palustris* [11, 12]. According to structural studies, esterases have a canonical *α*/*β*-hydrolase fold, which is composed of a central β-sheet surrounded by α-helices. The active site contains a catalytic triad formed by Ser-Asp-His residues, which is also found in other esterases and serine proteases. In addition, their enzymatic reaction mechanisms using this catalytic triads have been well studied [13, 14].

Over the last few years, many esterases with different enzymological properties and substrate specificities have been isolated from psychrophilic microorganisms including *Psychrobacter cryohalolentis* K5^T^, *Pseudomonas mandelii*, *Psychrobacter pacificensis*, and *Thalassospira* sp. GB04J01 [15–18]. Psychrophilic organisms living in a permanently cold environment produce enzymes adapted to function and display high catalytic efficiency at low temperatures [19, 20]. The relatively high activity of cold-active enzymes in low temperatures can be explained by two factors. The first is a decrease in the enthalpy of these enzymes due to a reduction in protein-ligand interactions. Thus, this may allow for substrate-binding and product-release with a low energy barrier at low temperatures. The second factor is an increase in the entropy difference between the apo-enzyme and the enzyme-substrate complex, due to the conformational flexibility of cold-active enzymes during substrate binding. It is known that these two factors cooperatively induce favorable reactions in cold-active enzymes at low temperatures [21, 22]. With ongoing demand for reducing energy consumption, cold-adapted esterases have great commercial potential and offer efficient catalytic alternatives for industrial applications [23]. Thus far, relatively fewer esterases from psychrophilic organisms have been studied compared with those from their mesophilic counterparts.

In this study, we report on the detailed structural and biochemical characteristics of an esterase (*Ea*EST) from a psychrophilic bacterium *Exiguobacterium antarcticum* B7 [24]. This organism was isolated from a microbial biofilm at Ginger Lake on King George Island, Antarctic Peninsula. We determined the crystal structure of *Ea*EST complexed with a peracetate molecule, which indicates that *Ea*EST has perhydrolase activity. Perhydrolysis may be a side activity of esterases and lipases [25–27]. However, it can be useful in industry and organic synthesis. In our activity assay, *Ea*EST demonstrated significant perhydrolysis activity with a *k*_cat_ value of 0.24 ± 0.01 s^-1^, which is higher than that of the wild-type esterase (0.12 ± 0.01 s^-1^) from *Pseudomonas fluorescens* (P*f*EST). Recent studies on *Pf*EST revealed that a single mutation, L29P, increased the perhydrolase activity to 5.1 ± 0.4 s^-1^ [27]. Therefore, *Ea*EST can also provide a good template for constructing a perhydrolase with a higher activity via protein engineering.

The crystal structure of peracetate-bound *Ea*EST was resolved at high resolution, revealing that *Ea*EST retains the characteristic *α*/*β*-hydrolase fold with a cap domain and has a catalytic triad of Ser96, Asp220, and His248. The optimal pH and temperature of *Ea*EST, as well as its substrate profiles and enantioselectivity, were investigated through mutational analysis. We found that *Ea*EST has broad substrate profiles including short-chain p-nitrophenyl esters (≤C8), naphthyl derivatives, and glyceryl tributyrate. Furthermore, the resulting immobilization of *Ea*EST showed significantly enhanced stability and reusability. Our results will provide a platform for the rational design and engineering of more robust biocatalysts.

## Materials and Methods

### Cloning and protein expression of *Ea*EST

The *Ea*EST gene (GenBank Accession CP003063) was amplified from chromosomal DNA of *E*. *antarcticum* B7 (Korea Collection for Type Cultures (KCTC), Republic of Korea) by PCR (hot start at 94°C (10 min) followed by 94°C (1 min), 58°C (45 sec), and 72°C (1 min), for 30 cycles with appropriate primers). The resulting PCR product was inserted into a pET-21a vector (Novagen, WI, USA), and the recombinant construct (pET-*Ea*EST) was transformed in *E*. *coli* BL21 (DE3). A single colony was inoculated into LB medium containing ampicillin (100 mg/ml) until the optical density at 600 nm (OD_600_) reached 0.4~0.6. Then, induction was done with the addition of 1 mM isopropyl-β-D-1-thiogalactoside (IPTG) for 3 h. The resulting cells were harvested, sonicated, and resuspended in a lysis buffer containing 20 mM Tris-HCl, pH 8.0, 10 mM imidazole, and 100 mM NaCl. After centrifugation at 18,000 rpm for 1 h, cell lysates were loaded onto a His-Trap Ni-NTA column using an AKTA Prime Plus (GE Healthcare, Little Chalfont, U.K.). Finally, after extensive washing with a buffer containing 20 mM imidazole, the bound *Ea*EST was eluted with an elution buffer containing 250mM imidazole. The eluted proteins were desalted using PD-10 column with 20 mM Tris-HCl (pH8.0) containing 100 mM NaCl. The protein purity of *Ea*EST was confirmed by SDS-PAGE and one liter of culture of the transformed *E*. *coli* usually resulted in approximately 8 mg of *Ea*EST.

### Crystallization of *Ea*EST

*Ea*EST was purified and concentrated to 24.4 mg/mL in a buffer consisting of 20 mM Tris-HCl pH 8.0 and 100 mM NaCl. A preliminary crystallization screen was performed with a mosquito robot (TTP Labtech, UK) using commercially available screening kits such as MCSG1-4 (Microlytic) and Index (Hampton Research). Initial screening was performed using sitting-drop vapor-diffusion method in 96-well crystallization plates at 293K. Two-hundred nanoliters of *Ea*EST was mixed with an equal volume of reservoir solution and equilibrated against 80 μL of reservoir solution. Initial crystals were obtained after 1 day under several different conditions. The hexagonal shaped single crystal was obtained from 0.2 M ammonium acetate, 0.1 M HEPES:NaOH pH 7.5, and 25% (w/v) PEG 3350 (MCSG2 #27). For further optimization, hanging-drop vapor-diffusion method was used in 24-well crystallization plates. Drops consisting of 1 μL of *Ea*EST and 1 μL of reservoir solution were equilibrated against 500 μL of reservoir solution. Optimized single crystals appeared after 1 day of incubation with 0.2 M ammonium acetate, 0.1 M HEPES:NaOH pH 7.5, and 27% (w/v) PEG 3350 at 293K.

### Data collection and structure determination of *Ea*EST

A single crystal of *Ea*EST was harvested and transferred to Pratone-N oil for cryoprotection. A data set containing 180 images at the resolution of 1.90 Å was collected on a 7A beam line at Pohang Accelerator Laboratory (PAL; Pohang, Korea) at 100K. The diffraction data were indexed, integrated, and scaled using the program HKL-2000 [28]. An *Ea*EST crystal belongs to the trigonal space group *P*3, with unit cell parameters of a = 76.765 Å, b = 76.765 Å, c = 68.161 Å, and α = β = 90°, γ = 120°. The volume of the asymmetric unit allows the presence of two copies with a Matthews coefficient of 1.83 Å^3^Da^-1^ and a solvent content of 32.84% [29]. The structure of *Ea*EST was determined by molecular replacement using the program MOLREP [30]. The coordinates of an esterase from *P*. *fluorescens* (PDB cold 3HI4) [27], which has 39% identity to *Ea*EST, was used as a model for molecular replacement. The resulting coordinate was rebuilt and refined manually based on electron-density maps using the programs REFMAC5 and COOT [31, 32]. After multiple rounds of structural refinement, the final structure of *Ea*EST has the R_work_ and R_free_ of 0.185 and 0.238, respectively, with a total of 542 amino acid residues and 191 water molecules. The statistics of data collection and structure refinement are listed in Table 1. The atomic coordinates and structure factors have been deposited in the Protein Data Bank (http://www.rcsb.org/) under accession code 5H3H.

**Table 1:** X-ray diffraction data collection and refinement statistics.

**Data set**	***Ea*EST complexed with peracetate**
X-ray source	Beam line 7A, PAL
Space group	*P*3
Wavelength (Å)	0.97933
Range of resolution (Å)	50.00–1.90 (1.93–1.90)
No. of observed reflections	172600
No. of unique reflections	35239 (1780)
*R*_merge_ ^a^	0.096 (0.500)
CC_1/2_	0.97(0.88)
Average *I/σ*	43.0 (16.0)
Completeness (%)	99.3 (100.0)
Redundancy	4.9 (5.5)
**Refinement**	
Resolution (Å)	50.01–1.90 (1.95–1.90)
No. of reflections in working set	33467 (2463)
No. of reflections in test set	1768 (120)
No. of residues	542
No. of water molecules	191
*R*_cryst_ ^b^ total	0.185 (0.200)
*R*_free_ ^c^ total	0.238 (0.282)
R.m.s. bond length (Å)	0.018
R.m.s. bond angle (°)	2.00
Average B value (Å^2^) (protein)	28.432
Average B value (Å^2^) (solvent)	32.753

^a^*R*_sym_ = ∑ | <*I*> − *I* | /∑<*I*>.

^b^*R*_cryst_ = ∑ | |*Fo*| − |*Fc*| | /∑|*Fo*|.

^c^*R*_free_ calculated using high-resolution data with 10% of all reflections excluded from refinement stages.

Values in parentheses refer to shells at highest resolution.

### Analytical ultracentrifugation (AUC)

Sedimentation velocity data were obtained at 20°C using a XL-A analytical ultracentrifuge (Beckman Coulter). *Ea*EST protein (2 mg/ml) in 25mM Tris-HCl pH 8.0, 200mM NaCl, 5 mM MgCl_2_ and 2 mM dithiothreitol (DTT) and the reference butter were loaded into a dual sector Epon centerpiece. *Ea*EST was spun at 45,000 rpm and the movement of a boundary formed by high centrifugal force was monitored over time at the wavelength of 280 nm. The obtained data was analyzed and calculated *c*(*s*) using the program SEDFIT [33, 34].

### Enzyme assay and immobilization of *Ea*EST

For esterase activity of *Ea*EST, *p*-nitrophenyl esters of different acyl chain lengths including *p*-nitrophenyl acetate (C_2_, *p*-NA), butyrate (C_4_, *p*-NB), hexanoate (C_6_, *p*-NH), octanoate (C_8_, *p*-NO), decanoate (C_10_, *p*-NDec), dodecanoate (C_12_, *p*-NDo), and phosphate *(p*-NP) were used as substrates. The release of *p*-nitrophenol was measured at 405 nm using an EPOCH2 microplate reader (Biotek, USA). Regioselectivity of *Ea*EST was also studied by using 1-naphthyl phosphate (1-NP), 1-naphthyl acetate (1-NA), 1-naphthyl butyrate (1-NB), and 2-naphthyl acetate (2-NA). The absorbance was measured at 310 nm.

The kinetic constants of *Ea*EST toward acetic acid perhydrolysis were measured using a monochlorodimedone (MCD) assay at 25°C. All reactions contained 0.047 mM of MCD, 149 mM of potassium bromide, and appropriated amounts of the enzyme. The concentrations of acetic acid were varied to 1.4 M. The reaction was initiated with the addition of 9.9 mM of hydrogen peroxide. Enzyme activity was determined by the halogenation of MCD (ε = 19.9 mM^−1^ cm^−1^ at 290 nm) as described previously [35]. The data were fit to the Michaelis-Menten equation using nonlinear regression (GraphPad Prism 5 Software, San Diego, CA, USA).

The optimal temperature and pH were investigated in the assay mixture containing 20 mM Tris-HCl, 100 mM NaCl (pH 8.0), 0.5 mM *p*-NA, and 10 μg of *Ea*EST. The optimal pH was studied by measuring enzyme activity of *Ea*EST from pH 3.0 to pH 10.0 at 25°C. Following buffers were used including 50 mM citrate-NaOH (pH 3.0–6.0), 100 mM phosphate-NaOH (pH 7.0), 50 mM Tris-HCl (pH 8.0), and 20 mM glycine-NaOH (pH 9.0–10.0). The optimal temperature was examined at 20, 40, 45, 50, 55, 60, and 80°C. Thermostability of *Ea*EST was measured by incubating the enzyme at 0, 20, 40, 50, and 60°C for 1 h. Each aliquot was taken every 15 min for measuring the residual activity.

The effects of NaCl and glycerol additions on *Ea*EST were determined by incubating the enzyme with various concentrations of NaCl (0–5 M) or glycerol (0–5 M) at 25°C for 1 h. For chemical stability of *Ea*EST, the effects of ethanol, isopropanol (i-PrOH), SDS, Tween 20, Triton X-100, and phenylmethylsulfonyl fluoride (PMSF) were determined. For enantioselectivity analysis, a pH shift-colorimetric assay was carried out with (*R*)- and (*S*)-methyl-3-hydroxy-2-methylpropionate in 20 mM Tris-HCl (pH 8.0), 100 mM NaCl in 100 μl reaction mixture. The absorbance spectra were recorded from 350 nm to 600 nm. This pH shift-colorimetric assay was also used for the hydrolysis of phenyl acetate, 2-phenylethyl acetate, and 2-methylbutyl acetate. In addition, the hydrolysis of glyceryl tributyrate, glyceryl trioleate, olive oil, and fish oil were measured with this assay. Fluorescence analysis was executed using a Jasco FP-8200 spectrofluorometer (MD, USA). *Ea*EST samples were incubated with different concentration urea (0–5 M) for 1 h. After excitation at 280 nm, emission spectra were recorded from 300 nm to 400 nm using a 5 nm slit width and a scan speed of 250 nm/min.

To prepare immobilized forms of *Ea*EST, a purified *Ea*EST (2 mg) was precipitated with 80% ammonium sulfate and crosslinked with 50 mM glutaraldehyde with gentle inverting for 12 h. Then, suspension was centrifuged at 13,000 rpm at 4°C for 30 min and the resulting immobilized *Ea*ESTs were washed 3 times with 20 mM Tris-HCl (pH 8.0), 100 mM NaCl. Activity of immobilized *Ea*EST was monitored by measuring the hydrolysis of *p*-nitrophenyl acetate (C_2_, *p*-NA). Thermal stability of immobilized *Ea*EST was investigated at 80°C and the activity of soluble *Ea*EST was set to 100%. To examine reusability, immobilized *Ea*EST was retrieved by simple centrifugation after each reaction. After repeated washing steps (usually 3 times), new substrate was added for another cycle and the activity of immobilized *Ea*EST was measured. For surface morphology of immobilized *Ea*EST, a scanning electron microscope (SUPRA 55VP, Carl Zeiss, Jena, Germany) was used. Samples were prepared by fixation process with 0.05 M cacodylate buffer (pH 7.2) containing 1% osmium tetraoxide (OsO_4_) at 4°C and consecutive cycles of dehydration by ethanol solutions. After drying with hexamethyldisilazane solution, samples were mounted on metal stubs and sputtered with gold.

## Results and Discussion

### Overall structure of *Ea*EST

**Fig 1:**
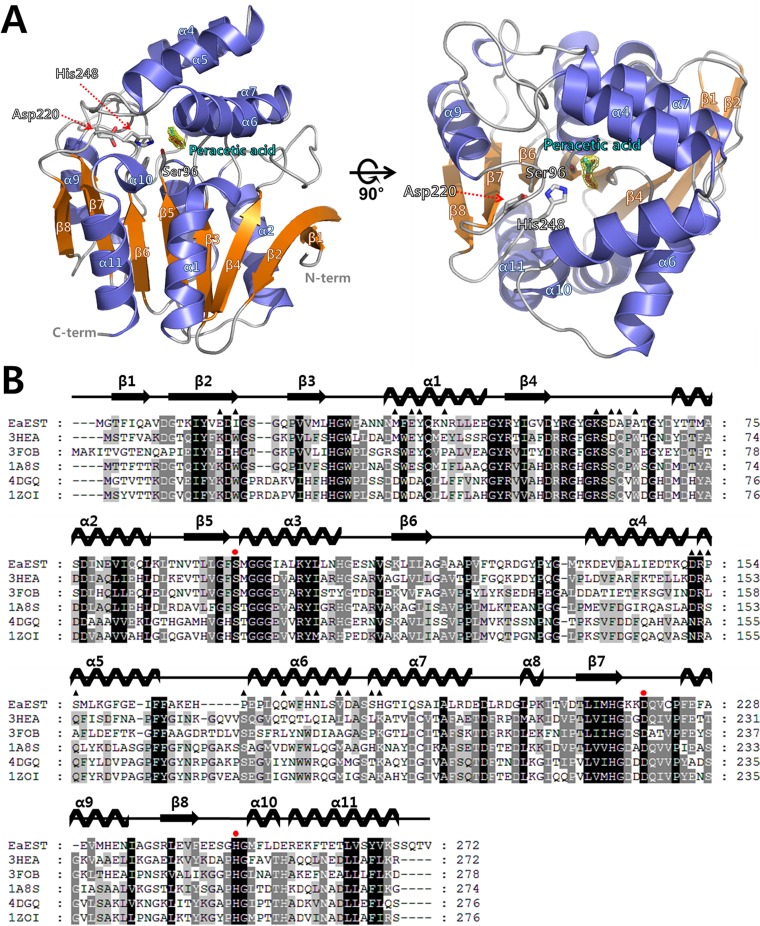
Crystal structure of peracetate-bound *Ea*EST. (**A**) Overall structure of monomeric *Ea*EST is shown in front view (left panel) and top view (right panel) rotated at 90°. The structure of *Ea*EST was drawn as a ribbon diagram with α-helices colored slate blue and β-strands colored orange. Bound peracetate molecule (cyan) is shown as a stick model with a 2Fo-Fc electron-density map (green) at 1.0 σ. The catalytic triad residues (Ser96, Asp220, and His248) are shown as stick models. *N*- and *C*-termini are labeled in gray letters and indicated with a red dashed arrow. (**B**) Multiple sequence alignment of *Ea*EST (NCBI reference sequence number WP_014970431.1), aryl esterase (PDB code 3HEA; UniProtKB code P22862), bromoperoxidase (PDB code 3FOB; UniProtKB code Q81NM3), haloperoxidase (PDB code 1A8S; UniProtKB code O31158), chloroperoxidase (PDB code 4DGQ; UniProtKB code B4EA96), and esterase (PDB code 1ZOI; UniProtKB code Q3HWU8). Secondary structural elements in the crystal structure of *Ea*EST are represented above the multiple sequence alignment. The catalytic triad residues (Ser96, Asp220, and His248) are indicated with red circles and the residues involved in the trimer interaction are indicated by black triangles. The multiple sequence alignment was performed with *ClustalX* and edited with *GeneDoc*.

**Fig 2:**
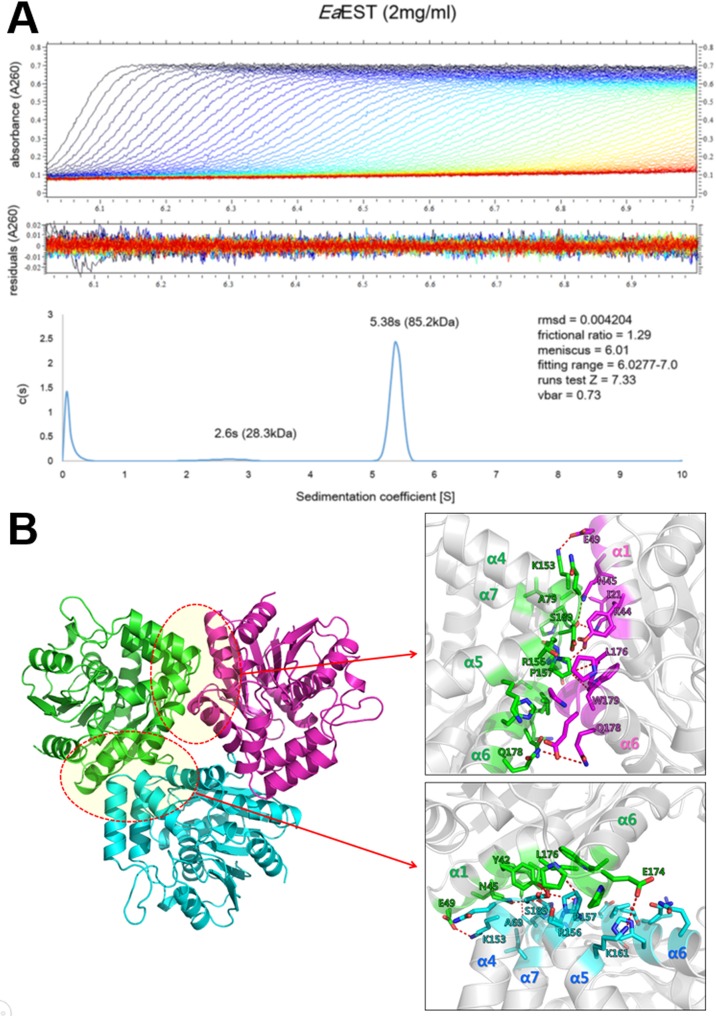
Trimeric structure of *Ea*EST. (A) Analytical ultracentrifugation (AUC) experiments using 2 mg/mL *Ea*EST give a mass of 85.2 kDa (sedimentation coefficient of 5.38 S and a frictional ratio of 1.29), indicating that *Ea*EST is a stable trimer in solution. (B) *Ea*EST trimer has a triangular shape. Each protomer has two binding interfaces for trimerization.

The crystal structure of an esterase (*Ea*EST) from *Exiguobacterium antarcticum* was determined in a *P*3 space group. As a search model, we used the structure of aryl esterase (*Pf*EST, PDB code 3HI4) from *Pseudomonas fluorescens* with a molecular replacement (MR) method. The final model was refined up to 1.9 Å and produced R_work_ and R_free_ values of 0.185 and 0.235, respectively (Table 1). The structure of an *Ea*EST monomer is composed of 11 α-helices and eight β-strands with dimensions of 45 × 42 × 40 Å. The structure shows a typical αβ-hydrolase fold with an α-helical cap domain. The central eight β-strands are surrounded by seven side α-helices (α1-α3 and α8-α11) and an α-helical cap domain (α4-α7 helices) (Fig 1). The asymmetric unit of an *Ea*EST crystal contains two protomers. The results of analytical ultracentrifugation demonstrated that the purified *Ea*EST protein is a stable trimer in solution (Fig 2A). Additionally, a crystallographic symmetry operator generates a tight trimeric arrangement, as shown in Fig 2B. The trimer interface in *Ea*EST is formed by α1, the β4-α2 loop region, α4, α5, α6, and an α7-helix (Figs 1 and 2B). Using mainly hydrogen bonds and hydrophobic interactions, the trimer interface obscures 466 Å^2^ of the total monomer surface area (3176 Å^2^).

A search of structural homologs using the DALI server identified an aryl esterase (PDB code 3HEA and 3HI4) as the closest structural homolog of *Ea*EST; additionally, bromoperoxidase (PDB code 3FOB), haloperoxidase (PDB code 1A8S), chloroperoxidase (PDB code 4DGQ), and esterase (PDB code 1ZOI) were included in the top five hits (Table 2) [36]. The top solution structure was the *Pf*EST structure used in the MR search; the *Pf*EST structure was aligned to the *Ea*EST structure with 0.96 Å r.m.s. deviation for 307 C_α_ atoms. Notably, all of the listed proteins in DALI result from trimer in solution; the exception to that is haloperoxidase (PDB code 1A8S), the oligomeric state of which has not yet been experimentally resolved.

**Table 2:** Selected structural homologs of *Ea*EST obtained using DALI search (DALI-Lite server).

Protein	PDB code	DALI score	Biological unit	Sequence % ID with *Ea*EST (aligned residue number/total number of residues)	Reference
aryl esterase	3HEA	42.7	Trimer	38% (266/271)	[27]
bromoperoxidase	3FOB	41.3	Trimer	34% (267/276)	It has not yet been published
haloperoxidase	1A8S	41.3	^a^ND	33% (266/273)	[37]
chloroperoxidase	4DGQ	40.5	Trimer	38% (266/271)	It has not yet been published
esterase	1ZOI	39.8	Trimer	25% (266/275)	[38]

^a^ND means not determined experimentally.

**Fig 3:**
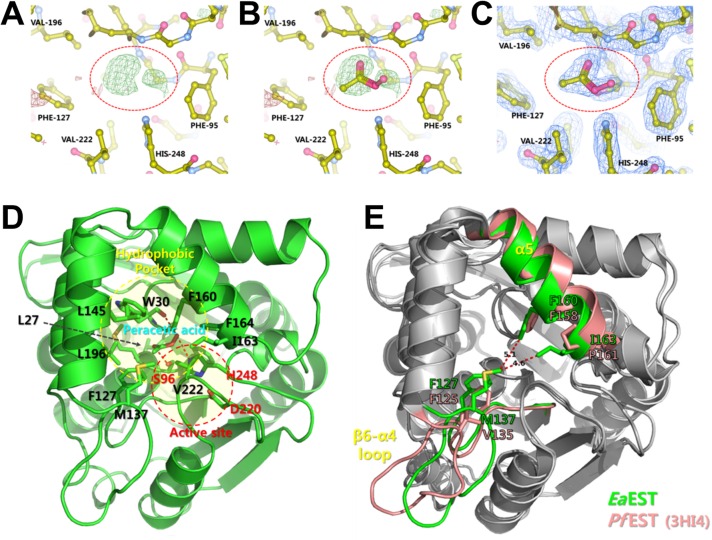
Peracetate-binding in *Ea*EST. (A) The positive electron density (green) was observed in the Fo-Fc omit map (contoured at 2.0 σ). (B) The peracetate model was overlaid on the Fo-Fc omit map (green, contoured at 2.0 σ). (C) The 2Fo-Fc electron-density map at 1.0 σ is shown after peracetate model building and refinement. (D) Peracetate-binding site is shown. Side chains of residues near ligand recognizing hydrophobic pocket (yellow circle) and active site (red circle) are indicated by sticks. (E) Conformational changes between peracetate-bound *Ea*EST and acetate-bound *Pf*EST (PDB code 3HI4). The β6-α4 loop region of *Ea*EST may undergo conformational change to open the entrance hydrophobic channel for ligand exchange.

During the process of structure determination, unknown electron-density maps were found near the active sites of both molecules in the asymmetric unit (Fig 3A). Even if no specific ligands were added to *Ea*EST at purification and crystallization, the unknown electron density was initially interpreted as an acetate because of crystallization conditions containing 0.2 M ammonium acetate. After building and refining the model, additional electron density was clearly observed in the hydroxyl group of acetic acid, indicating that *Ea*EST has perhydrolysis activity via acetic acid. Therefore, peracetic acid could be located. Using repetitive refinement, the peracetate molecule was perfectly fitted into the electron-density map (Fig 3B). The active site of *Ea*EST is comprised of a conserved catalytic triad (Ser96, Asp220, and His248) and the neighboring hydrophobic pocket, which is composed of the hydrophobic residues Trp30, Phe127, Met137, Leu145, Met156, Phe160, Ile163, Leu199, Phe164, and Val 222. The hydrophobic pocket may play a role in portal substrate entry and initial binding. The hydroxyl group of peracetic acid forms hydrogen bonds with the O atom of Ser96, the NE2 atom of His248, and main-chain oxygen atom of Trp30. Additionally, methyl group of peracetic acid toward the hydrophobic pocket of active site (Fig 3B and 3C).

### Comparison of *Ea*EST structure with that of *Pf*EST

**Fig 4:**
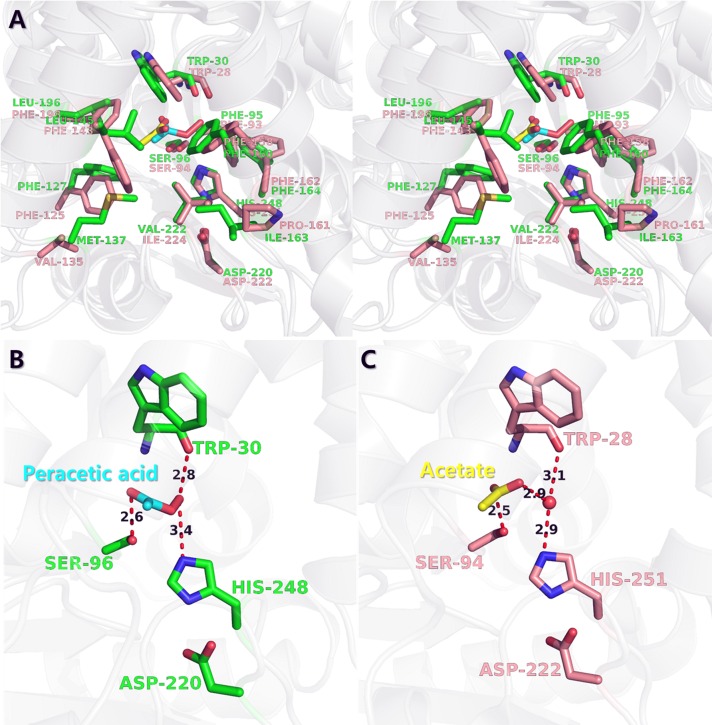
Structural comparisons of ligand-binding site between peracetate-bound *Ea*EST and acetate-bound *Pf*EST. (A) Stereo view of the superimposed structure of peracetate (cyan)-bound *Ea*EST (green) and acetate (yellow)-bound *Pf*EST (PDB code 3HI4, acetate-bound form, salmon). The residues comprising the active and ligand-binding sites are shown in a stick representation. (B) Peracetate-binding mode and its interactions in *Ea*EST structure. (C) Acetate-binding mode and its interactions in *Pf*EST structure.

Structural comparison using superimposition between *Ea*EST and *Pf*EST (PDB code 3HI4, acetate-bound form) shows that the overall monomer structures, as well as trimeric structures, are very similar, but the local orientation of the loop region between β6 and α4 (residues 123–141) differs slightly. In the *Ea*EST structure, the β6-α4 loop protrudes inward, toward the center of the hydrophobic pocket, located at the vicinity of the active site. The Met137 residue, located on the β6-α4 loop, enables hydrophobic interactions with Ile163, Leu145, Phe127, and Phe160. Similarly, the Phe127 residue, located on the β6-α4 loop, is also oriented toward the hydrophobic pocket, more than is the Phe125 of *Pf*EST. These interactions also induce further movement of the α5-helix toward the β6-α4 loop region. The α5-helix is directly involved in substrate interactions (Fig 3C). A comparison of binding sites revealed that the β6-α4 loop region also plays an important role in substrate binding. The catalytic triad of Ser96, Asp220, and His 248 (Ser94, Asp222, and His251 in *Pf*EST) is conserved, but the residues of the neighboring hydrophobic pocket show clear distinctions. Notably, the Met137 residue (Val135 in *Pf*EST), located on the β6-α4 loop region, shows the largest structural difference (Figs 3D and 4A). In *Pf*EST, the Val135 residue, located on the β6-α4 loop region, shows a relatively open state of its active site. In *Ea*EST, however, The Met137 residue extends to the hydrophobic pocket and shows a closed state compared with that of *Pf*EST. Because of these interactions, peracetate may bind to the active site of *Ea*EST more tightly and deeply than acetate binds to the active site of *Pf*EST. Peracetate interacts with Trp30, Ser96, and His248 directly. In *Pf*EST, acetate interacts with Trp28 and His251 via mediation by a water molecule. The protruding hydroxyl group of peracetic acid substitutes for a water molecule (Fig 4B and 4C). Therefore, these investigations suggest that the β6-α4 loop region may be critical for controlling ligand recognition and may function as a selective substrate filter.

### Functional analysis of *Ea*EST

**Fig 5:**
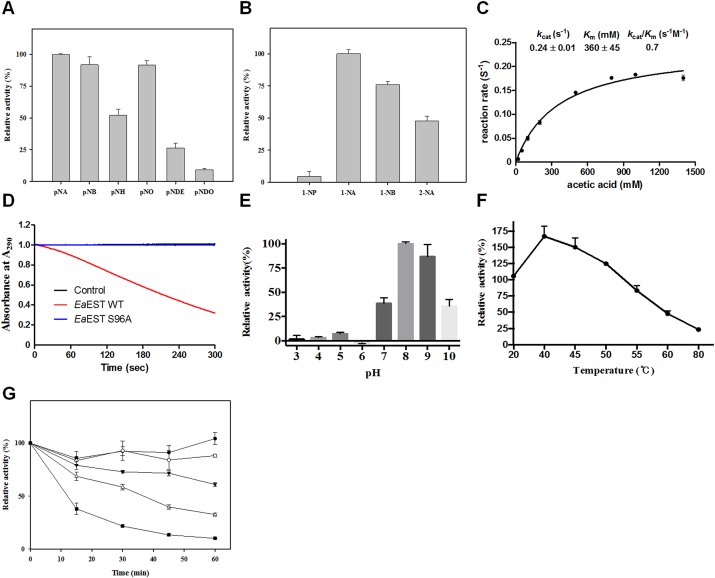
Enzyme activity of *Ea*EST. (A) The substrate profiles of *Ea*EST were determined using different *p*-NP esters (A) and naphthyl derivatives (B). (C) The reaction rate of acetic acid perhydrolysis catalyzed by wild-type *Ea*EST. Perhydrolase activities were measured at pH 5.5 at 25°C. Kinetic constants were obtained by varying the concentration of acetic acid. (D) Specific activities for acetic acid perhydrolysis catalyzed by wild-type *Ea*EST and the S96A mutant. (E) pH stability of *Ea*EST. The pH dependence of hydrolysis of *p*-NA by *Ea*EST was measured at 25°C. (F) Effect of temperature on the residual activity of *Ea*EST. (G) Thermostability of *Ea*EST. Residual activities are expressed relative to the original activity during incubation at 0, 20, 40, 45, and 50°C. The results are the mean of three individual experiments.

The substrate profiles of *Ea*EST were investigated using *p*-nitrophenyl esters such as *p-*NA, *p*-NB, *p-*NH, *p-*NO, *p*-NDec, and *p*-NDo. The enzyme showed considerable activity toward all tested substrates except for *p*-NDo (Fig 5A). The highest activity was observed with *p*-NA, *p-*NB, and *p*-NO, whereas the hydrolytic level of *p*-NDo was 90% lower than that of *p*-NA (set at 100%). We also analyzed substrate profiles of *Ea*EST using naphthyl derivatives. As shown in Fig 5B, *Ea*EST showed the highest activity toward 1-NA, followed by 1-NB and 2-NA, but showed low activity toward 1-NP.

Previously, *Pf*EST and its mutant showed perhydrolase activity, the reversible formation of peracetic acids from acetic acids and hydrogen peroxide, as well as esterase activity [26, 27]. The findings of our structural study led us to hypothesize that peracetate can bind to the active site of *Ea*EST. Thus, in order to clarify whether *Ea*EST also had perhydrolase activity, kinetic constants for acetic acid perhydrolysis were measured using the MCD assay with varied concentrations of acetic acid and 9.9 mM hydrogen peroxide. We found that *Ea*EST is a good catalyst for perhydrolysis of acetic acid, showing a high *k*_cat_ value of 0.24 ± 0.01 s^-1^ compared with that of *Pf*EST (0.12 ± 0.02 s^-1^) (Fig 5C). Mutant S96A showed very low perhydrolase activity toward acetic acid (Fig 5D).

The optimum pH and temperature of *Ea*EST was monitored at the pH range of 3.0 to 10.0 and temperature range of 20 to 80°C using *p*-NA as substrate. *Ea*EST exhibited maximum activity at pH 8.0–9.0. However, the enzyme was rapidly inactivated at below pH 6.0, with less than 10% of its maximum activity remaining (Fig 5E). The optimum temperature for the activity of *Ea*EST was 40°C (Fig 5F). The effect of temperature on the stability of *Ea*EST was investigated by measuring the residual activity of *Ea*EST at 15 min intervals using a temperature range of 0 to 50°C. There was a gradual reduction in activity as temperature increased. *Ea*EST retained more than 50% of its initial activity after incubation at 45°C for 30 min, while a significant loss of activity occurred after only 15 min when the enzyme was incubated at 50°C (Fig 5G).

**Fig 6:**
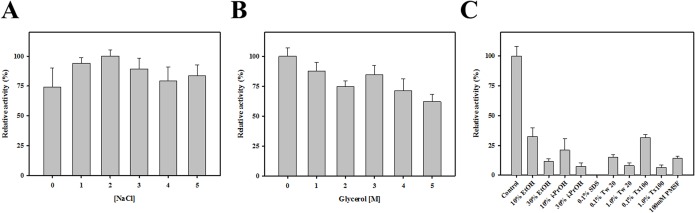
**Effect of NaCl (A) and glycerol (B) on the activity of *Ea*EST**. Relative activity was determined by incubating the enzyme with different concentrations of NaCl and glycerol (0 to 5 M). The maximum activity value obtained was set to 100%. (C) Chemical stability of *Ea*EST. Residual activity after 1 h of incubation is expressed relative to the original activity obtained without the addition of chemical compounds (100%).

Next, we investigated the effects of NaCl and glycerol on the function of *Ea*EST. The enzyme was most active in the presence of 2 M NaCl, but concentration of NaCl did not significantly affect enzyme activity (Fig 6A). Although, the activity decreased with increasing glycerol concentration, more than 60% of maximal activity was retained in the presence of 5 M glycerol (Fig 6B). To examine the effects of detergents and organic solvents on the stability of *Ea*EST, the enzyme was incubated with each chemical compound, and then residual activities were measured. The results show that all the chemicals used inhibited the activity of *Ea*EST, with less than 40% of its original activity remaining. Among these chemicals, sodium dodecyl sulfate (SDS) at 1.0% (w/v) completely inhibited the activity of *Ea*EST (Fig 6C).

### Enantioselectivity analysis and activity assay of *Ea*EST and S96A mutant

**Fig 7:**
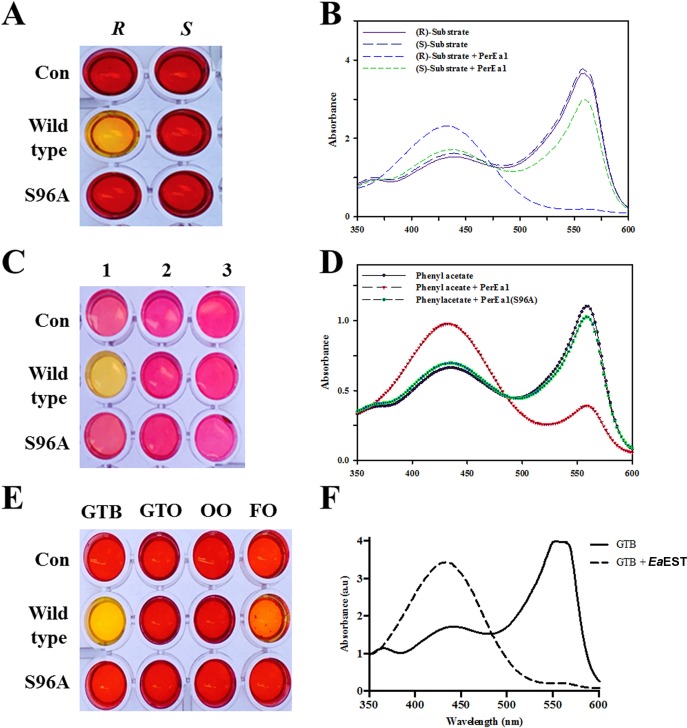
(A) pH shift assay for enantioselectivity analysis of *Ea*EST and S96A mutant conducted with (*R*)- and (*S*)-methyl-3-hydroxy-2-methylpropionate. (B) Absorbance spectra of the reaction mixtures in (A). (C) Hydrolysis of phenyl-substituted substrates: 1, phenyl acetate; 2, 2-phenylethyl acetate; 3, 2-methylbutyl acetate. (D) Absorbance spectra of 1 from (C) were measured. (E) Hydrolysis of glyceryl esters (glyceryl tributyrate [GTB] and glyceryl trioleate [GTO]) and oils (olive oil [OO] and fish oil [FO]) by *Ea*EST was investigated. (F) Absorbance spectra of GTB hydrolysis by wild-type *Ea*EST were measured.

Carboxylesterase has become increasingly attractive due to its ability to perform enantioselective biotransformation in the production of chiral pharmaceuticals. Thus, we conducted an enantioselectivity analysis of *Ea*EST using a pH shift assay with (*R*)- and (*S*)-methyl-3-hydroxy-2-methylpropionate. Hydrolytic activity was detected based on color and changes in absorbance. As shown in Fig 7A and 7B, a change of color to yellow was observed only in the mixture containing the (*R*)-enantiomer, which indicates that *Ea*EST prefers the (*R*)-enantiomer of the chiral ester to the (*S*)-enantiomer. The result was confirmed by measuring the absorbance spectra. When we performed these assays using the catalytic-site mutant S96A, the mutant showed no detectable enzymatic activity toward either the (*R*)- or the (*S*)-enantiomer. In addition to phenyl-substituted substrates, phenyl acetate, 2-phenylethyl acetate, and 2-methylbutyl acetate were also used to characterize *Ea*EST and the S96A mutant. The hydrolytic activity of *Ea*EST is limited to phenyl acetate. Only the reaction mixture containing phenyl acetate was observed to turn yellow, which is consistent with the results of the absorbance spectra (Fig 7C and 7D). Additionally, the enzymes were investigated for their ability to hydrolyze glyceryl esters and oils. We determined that *Ea*EST could hydrolyze glyceryl tributyrate but showed little or no activity toward glyceryl trioleate, olive oil, or fish oil. The S96A mutant had a severely reduced ability to hydrolyze the tested substrates (Fig 7E and 7F).

### Effect of urea on activity and conformation of *Ea*EST

**Fig 8:**
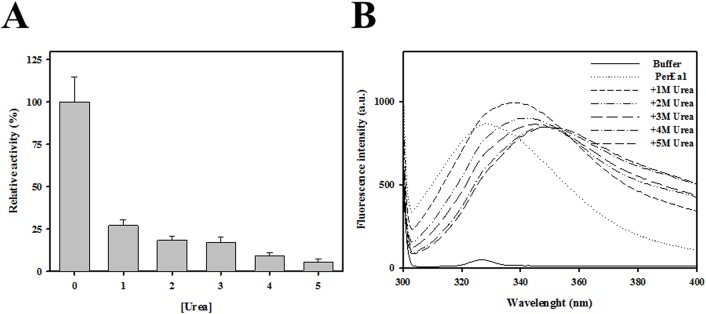
(A) Effect of concentration of urea on the activity of *Ea*EST. (B) Intrinsic fluorescence spectra were recorded with increasing concentrations of urea (0 to 5 M).

To explore the effects of urea on the activity of *Ea*EST, enzyme activity with respect to *p*-NA was measured in the presence of various concentrations of urea (Fig 8A). There was a significant reduction in activity at mildly denaturing condition of 1 M urea, with only 27% of the original activity retained. *Ea*EST activity decreased gradually with increasing urea concentrations, indicating that urea induced conformational changes at the active site of *Ea*EST. Further, to clarify the results of the activity assays, we monitored the intrinsic fluorescence emission spectra of *Ea*EST in the presence of different urea concentrations (Fig 8B). The conformational changes in *Ea*EST during urea-induced unfolding were monitored using fluorescence properties of tryptophan residues, which are highly sensitive to the environment. Under native conditions, the maximum fluorescence emission wavelength (λmax) of *Ea*EST is 327 nm. In 1 M urea, fluorescence intensity rapidly increased and λmax was red-shifted. However, increasing urea concentrations higher than 2 M caused a progressive decrease in fluorescence intensity, with accompanying red-shifted emission maximum ranging from 338 to 349 nm. The results can be attributed to the gradual exposure of Trp residues upon urea-induced unfolding of *Ea*EST.

### Biochemical analysis of the L27A mutant of *Ea*EST

**Fig 9:**
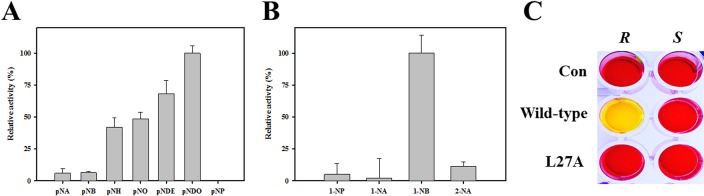
Analysis of substrate profiles and enantioselectivity of the L27A mutant. Substrate profiles of L27A were investigated toward different *p*-nitrophenyl esters (A) and naphthyl derivatives (B). (C) pH shift assay was conducted in the presence of (*R*)- or (*S*)-methyl-3-hydroxy-2-methylpropanoate.

For the mutagenesis study, we investigated substrate profiles and enantioselectivity of S96A and L27A, located in the catalytic site of *Ea*EST. Native gel analysis with purified wild-type and mutated *Ea*EST proteins indicated that L27A did not separate on the gel in contrast to wild-type and S96A. Moreover, the L27A mutant of *Ea*EST was expressed at a low level and this single mutation results in reduced solubility compared to the wild-type protein, suggesting that Leu27 may be important for protein solubility. Fig 9A shows substrate profiles of L27A for *p*-nitrophenyl esters. Interestingly, the replacement of Leu27 by Ala considerably changed the catalytic pattern of wild-type *Ea*EST. L27A showed the greatest preference for *p*-NDo and low-level activities toward *p*-NA and *p*-NB. Conversely, the wild-type was most active toward *p*-NA and *p*-NB, and did not prefer *p*-NDo. Substrate profiles of L27A for naphthyl derivatives were also investigated. Although L27A showed the highest activity against 1-NB, less than 20% of activity was obtained with 1-NP, 1-NA, and 2-NA relative to that of 1-NB. Wild-type *Ea*EST demonstrated substrate preference for 1-NA rather than for 1-NB, which is different from the preference of L27A (Fig 9B). Additionally, to examine whether this mutation can modulate enantioselectivity of *Ea*EST, pH shift assays were performed with (*R*)- and (*S*)-methyl-3-hydroxy-2-methylpropionate. As shown in Fig 9C, wild-type *Ea*EST showed preference for (*R*)-enantiomers, but the L27A mutant showed no activity toward either enantiomer. These results indicate that the Leu27 residue is critical for catalytic activity, as well as for protein solubility, of *Ea*EST.

### Immobilization of *Ea*EST

**Fig 10:**
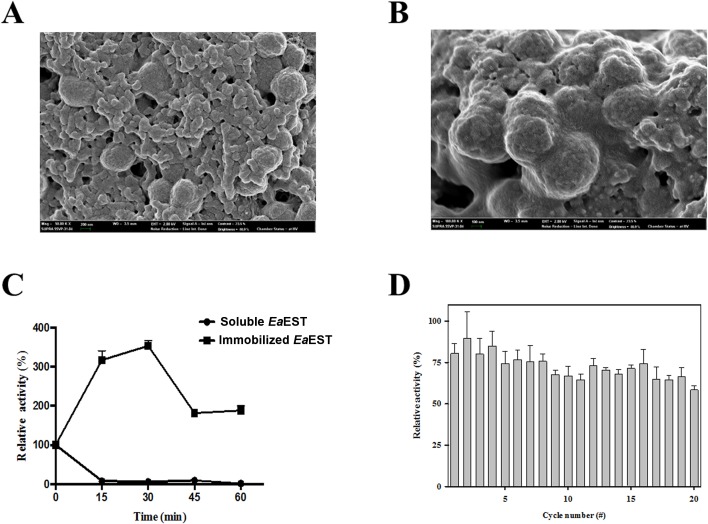
Immobilization of *Ea*EST. Scanning electron microscope (SEM) image of cross-linked enzyme aggregates (*Ea*EST-CLEAs). Representative images at 50 kX (A) and 100 kX (B) are shown. (C) Thermostability of immobilized *Ea*EST (■) and soluble *Ea*EST (●) at 80°C. Activity was measured every 15 min. (D) Reusability of immobilized *Ea*EST was compared to the soluble enzyme for 20 reaction cycles. Residual activities were expressed relative to those of free Sm23 (100%).

Enzyme immobilization is an effective strategy to improve the stability and recyclability of free enzymes. Immobilization of *Ea*EST was characterized for biotechnological and industrial applications. *Ea*EST was immobilized as a cross-linked enzyme aggregate (CLEA) via precipitation with ammonium sulfate and cross-linking with glutaraldehyde. Scanning electron microscopy (SEM) images of CLEAs show the morphological structure of the amorphous aggregate of *Ea*EST (Fig 10A and 10B). The thermostability of soluble and immobilized *Ea*EST was determined by pre-incubating at 80°C for various time intervals. Surprisingly, immobilized *Ea*EST exhibited significantly enhanced activity and stability. Approximately 350% of its initial activity was retained after exposure to 80°C for 30 min; even after incubating for 1 hour, immobilized *Ea*EST showed 200% of its initial activity. However, all of its soluble enzymatic activity was lost after only 15 min. The thermostability of the immobilized *Ea*EST was higher than that of the soluble enzyme (Fig 10C). Fig 10D shows reusability of immobilized *Ea*EST with *p*-NA hydrolysis. Immobilized *Ea*EST retained more than 60% of its initial activity after 20 reutilization cycles. The results suggest that immobilized *Ea*EST could be effectively reutilized for potential industrial applications.

## Conclusion

In this study, we characterized the *Ea*EST enzyme using structural analysis based on crystal diffraction and biochemical data and activity analysis. Using an analytical ultracentrifugation (AUC) analysis, we also confirmed that the purified *Ea*EST protein forms a stable trimer in solution. These studies provide insight into an unusual enzymatic feature, the dual catalytic role of *Ea*EST as a typical esterase and perhydrolase. The crystal structure of *Ea*EST in peracetate-bound form provides the first structural insights into the final product-bound state during perhydrolysis. A comparison of the structure of *Ea*EST with that of *Pf*EST (PDB code 3HI4) revealed that the bound peracetate has different interactions compared with the bound acetate in *Pf*EST. As a result, peracetate-binding induced rearrangements of the active site residues and conformational changes in β6-α4 loop region and the α-helix. In its esterase activity, *Ea*EST displays the broad substrate profiles for short-chain *p*-nitrophenyl esters (≤C8), naphthyl derivatives, phenyl acetate, and glyceryl tributyrate. Additionally, immobilized *Ea*EST showed approximately 200% of its initial activity and 60% activity after 20 reutilization cycles. Given that wild-type *Ea*EST has the dual activity of esterase and perhydrolase, it can be used as template in structure-based protein engineering for developing modified enzymes having different substrate specificities or a single robust activity. For example, our L27A mutant *Ea*EST showed different substrate preference and stereo selectivity compared with those of wild-type *Ea*EST.
